# IL-4 and IL-13 regulate TLR expression and eosinophil-basophil differentiation of cord blood CD34^+^ progenitor cells

**DOI:** 10.1186/1710-1492-10-S2-A60

**Published:** 2014-12-18

**Authors:** Pia Reece, Gail M Gauvreau, Roma Sehmi, Judah A Denburg

**Affiliations:** 1 Division of Clinical Immunology and Allergy, McMaster University, Hamilton, Ontario Canada, L8S 4K1; 2 Asthma Research Group, Firestone Institute for Respiratory Health, McMaster University Hamilton, Ontario, Canada L8N 4A6

## Background

Intrauterine environmental exposures have been shown to influence neonatal immunity and subsequent allergic disease development [1]. We have previously shown that cord blood (CB) progenitor cells of high atopic risk infants have reduced toll-like receptor (TLR) expression and produce fewer lipopolysaccharide (LPS)-stimulated eosinophil-basophil (Eo/B) colonies, compared to low-atopic risk infants. In the present study, we investigated whether a surrogate *ex vivo* T_H_2 milieu (i.e., either IL-4 or IL-13), could represent an underlying mechanism to explain our previous findings.

## Methods

CB CD34^+^ cells from healthy donors were cultured with IL-4 or IL-13 (in combination with LPS) and assessed for TLR-2, TLR-4, and TLR-9 expression using flow cytometry, as well as Eo/B differentiation using methylcellulose cultures. Pharmacological inhibitors were added to the methylcellulose cultures to determine the effect of blocking IL-4 or IL-13 signalling in CB CD34^+^ cells in relation to Eo/B colony forming unit (CFU) formation.

## Results

Stimulation of CD34^+^ cells with IL-4 or IL-13 trended to decreased expression of TLR-2 (p=0.063), whereas IL-4, but not IL-13, reduced Eo/B CFU formation in the presence of LPS. The latter was found to be dependent on IL-4Rα and not IL-13Rα1.

## Conclusions

Thus, the responsiveness of CB CD34^+^ progenitor cells to LPS is differentially regulated by the T_H_2 cytokines, IL-4 and IL-13, and may be related to TLR expression on these cells. Therefore, *in utero* interactions between placental-derived pro-allergic cytokines and neonatal progenitor cells influences CD34^+^ phenotype and function, with implications for Eo/B-mediated inflammatory responses in early life.
